# Recurrent flow patterns as a basis for two-dimensional turbulence: Predicting statistics from structures

**DOI:** 10.1073/pnas.2320007121

**Published:** 2024-05-31

**Authors:** Jacob Page, Peter Norgaard, Michael P. Brenner, Rich R. Kerswell

**Affiliations:** ^a^School of Mathematics, University of Edinburgh, Edinburgh EH9 3FD, United Kingdom; ^b^Google Research, Mountain View, CA 94043; ^c^School of Engineering and Applied Sciences, Harvard University, Cambridge, MA 02138; ^d^Department of Applied Mathematics and Theoretical Physics, Centre for Mathematical Sciences, University of Cambridge, Cambridge CB3 0WA, United Kingdom

**Keywords:** turbulence, dynamical systems, machine learning

## Abstract

A dynamical systems approach to turbulence envisions the flow as a trajectory through a high-dimensional state space [Hopf, *Commun. Appl. Maths* **1**, 303 (1948)]. The chaotic dynamics are shaped by the unstable simple invariant solutions populating the inertial manifold. The hope has been to turn this picture into a predictive framework where the statistics of the flow follow from a weighted sum of the statistics of each simple invariant solution. Two outstanding obstacles have prevented this goal from being achieved: 1) paucity of known solutions and 2) the lack of a rational theory for predicting the required weights. Here, we describe a method to substantially solve these problems, and thereby provide compelling evidence that the probability density functions (PDFs) of a fully developed turbulent flow can be reconstructed with a set of unstable periodic orbits. Our method for finding solutions uses automatic differentiation, with high-quality guesses constructed by minimizing a trajectory-dependent loss function. We use this approach to find hundreds of solutions in turbulent, two-dimensional Kolmogorov flow. Robust statistical predictions are then computed by learning weights after converting a turbulent trajectory into a Markov chain for which the states are individual solutions, and the nearest solution to a given snapshot is determined using a deep convolutional autoencoder. In this study, the PDFs of a spatiotemporally chaotic system have been successfully reproduced with a set of simple invariant states, and we provide a fascinating connection between self-sustaining dynamical processes and the more well-known statistical properties of turbulence.

## Significance

A long-standing challenge in turbulence has been to connect individual coherent structures to the more well-known statistical properties of the flow. Here, we establish such a connection by representing two-dimensional turbulence as a Markov chain between exact unstable periodic orbits, which are realized transiently in the flow. To find the dynamically relevant solutions, we develop a method based on optimization of a scalar loss function, which overcomes the restrictions of previous algorithms and is effective at a high Reynolds number. The Markovian representation is then achieved by using a neural network to label turbulent snapshots according to the nearest unstable solution, and its invariant measure reproduces full PDFs of the chaotic flow.

A compelling view of turbulence, originally advocated by Hopf (1), is to consider a turbulent flow as an orbit in a very high-dimensional state space. Turbulence is then considered as a transient realization of the unstable simple invariant solutions shadowed by this orbit as it “pinballs” between them (2, 3) This viewpoint is attractive for both mechanistic understanding, which can be found in the dynamics of the individual solutions, and for quantifying the relationship of individual dynamical events to long-time statistics. In recent decades, attempts to realize this approach have dramatically improved our understanding of transitional shear flows (4, 5): as examples, the onset of turbulence in pipes has been connected to the emergence of finite-amplitude traveling wave solutions beyond some critical Reynolds number, Re, in saddle node bifurcations (6, 7), while the later discovery of unstable periodic orbits (UPOs) in so-called “minimal” turbulent configurations (8, 9) has revealed some of the self-sustaining mechanisms at play in wall-bounded turbulence (10–12). However, extending these ideas to high-Re flows has been restricted by computational limitations both in the methods for finding UPOs and using them to label realizations of fully developed turbulence.

For fully developed turbulence the dynamical systems view imagines a state space littered with simple invariant solutions (equilibria, traveling waves, UPOs) whose entangled stable and unstable manifolds create the scaffold for the turbulent pinball (1, 6, 9, 13–19). This picture suggests a predictive theory based upon a suitably weighted sum of the properties of the visited invariant solutions where the weights reflect the relative time spent in the neighborhood of the solution (20). However, finding enough of the important solutions let alone identifying the appropriate weights has proved prohibitively expensive. This is especially so at high Re where the set of invariant solutions proliferates dramatically and each becomes increasingly unstable, hampering their identification.

A central and well-known issue is the sensitivity of the Newton–Raphson root-finding algorithm to the quality of the initial guess in high dimensional problems. Past work has mainly relied on “recurrent flow analysis” to generate good enough guesses for UPOs where a turbulent orbit is required to shadow a UPO for at least one full period (8, 9, 16, 18, 21, 22). In practice, this imposes a limit on how unstable a UPO can be, severely hampering the search as Re increases (16). A second issue is the strategy for recognizing when the flow has nearly recurred. Typically this is done simply with an Euclidean norm of the difference between initial and final states, and consequently, the threshold for near recurrence has to be set quite high. Finally, the third shortcoming is that many of the dynamically relevant periodic orbits are undiscovered, particularly those with a larger than average dissipation rate (16, 22). These difficulties have motivated a number of alternative approaches to guess generation (22–27) or even UPO identification (28) but none have been sufficiently transformational to demonstrate the connection between dynamics and statistics even in the simplest model problems of steady turbulence.

Even armed with a complete set of UPOs, a profound theoretical question is to predict the weights for how each UPO should be counted in a “UPO expansion” of the turbulent flow. Periodic orbit theory (20, 29–31) gives a theoretical prescription that is effective in low dimensional dynamical systems (e.g., ref. 3). Yet, applying this theory to the Navier–Stokes equations remains challenging. An early attempt to apply this theory to the 2D Navier–Stokes equations showed no greater skill than a control experiment of using equal weighting, although the set of solutions available was clearly too small (16, 22). At this point, even determining whether an expansion of an arbitrary turbulent flow in terms of UPOs is possible is an open question in fluid mechanics.

In this paper, we present computational approaches to both aspects of the problem which overcome many of the earlier limitations, introducing methods for both finding and converging UPOs and for defining weights by labeling turbulent data according to which solution is closest in state space. In contrast to earlier work, our method for UPO detection does not require careful construction of an initial guess, and yields large numbers of dynamically relevant UPOs. In order to do this, we adapt a recently developed fully differentiable flow solver (32), which allows us to find high-quality guesses for UPOs by performing gradient descent on a loss function involving entire solution trajectories. This allows explicit searches for periodic orbits with certain properties (e.g., high dissipation rates) and can successfully converge large numbers of UPOs starting from arbitrary turbulent snapshots. We then train deep neural networks to learn accurate low-order representations of the turbulence, which we are able to use to measure which UPO a turbulent orbit is closest to at any instant in time. The result is a Markovian turbulent dynamics, which not only allows us to define a set of weights for the UPOs via the chain’s invariant measure, but also yields insight into routes to extreme events. The weights discovered from the invariant measure generate robust reproduction of the statistics of the turbulent attractor, including the full dissipation PDF, realizing Hopf’s original picture. Although we develop these methods in a model problem, the underlying methodology promises to change our understanding of canonical flows at much higher Re.

## Periodic Orbit Search Strategy

### Two-Dimensional Turbulence.

We demonstrate our UPO search methodology within the widely studied turbulent “Kolmogorov” flow: monochromatically forced turbulence in a periodic domain taken here to be two-dimensional. The governing equations are

[1a] ∂tu+u·∇u=−∇p+1ReΔu+sin(ny)x^,

[1b] $$\begin{array}{cc} \nabla \cdot u & =0. \end{array}$$

where the Reynolds number is defined as $Re:=\sqrt{\chi }{\left(L_{y}^{*}/2\pi \right)}^{3/2}/\nu$, with χ the forcing amplitude and $L_{y}^{*}$ the height of the computational domain. The problem is nondimensionalized by the fundamental wavenumber in the vertical direction (hence the dimensionless $L_{y}=2\pi$), throughout $L_{x}=L_{y}$ is taken and we use the popular forcing wavelength n=4 (e.g., refs. 16, 22, 24, 27, 28, and 33). We also frequently use the out-of-plane vorticity, $\omega :=\left(\nabla \times u\right)\cdot \hat{z}$, both in the formulation for converging exact solutions (*SI Appendix*) and for training the neural networks used to label the solutions found. We consider two Reynolds numbers (Re=40 and Re=100) where self-sustaining turbulence is observed, with that at Re=100 clearly in the asymptotic regime (16). At Re=40 around 50 UPOs have been found previously, all with low average dissipation rates, while only 9 UPOs have been converged at Re=100 (16).

The governing equations [1] are equivariant under continuous shifts in the horizontal direction,

$$T^{\alpha }:\left[u,v,\omega \right]\left(x,y\right)\to \left[u,v,\omega \right]\left(x+\alpha ,y\right)$$

and consequently many of the simple invariant solutions are relative equilibria (traveling waves) or periodic orbits. There are also discrete shift-reflect (S:[u,v,ω](x,y)→[−u,v,−ω](−x,y+π/n), with $S^{2n}\omega =\omega$) and rotation-through-π (R:[u,v,ω](x,y)→[−u,−v,ω](−x,−y)) symmetries. Here, we seek relative unstable periodic orbits (RPOs) with some period T and shift α which satisfy

[2] $$\begin{array}{c} T^{\alpha }f^{T}\left(u\right)-u=0, \end{array}$$

where $f^{t}$ is the time-forward map of Eq. 1.

### Automatic Differentiation for Periodic Orbits.

We solve Eq. 1 (and the equivalent velocity-vorticity form described in *Materials and Methods*) using JAX-CFD, a fully differentiable flow solver where gradients of the time-forward map, $f^{T}\left(u\right)$, with respect to initial conditions can be computed via automatic differentiation to machine precision (32). This capability forms the basis of the periodic orbit search strategy. We make use of both the “standard,” finite-difference, primitive variable formulation (32) and the spectral, vorticity version (34) of JAX-CFD. The former is used to construct robust periodic orbit guesses, for reasons discussed below, and the latter for final convergence in a Newton solver and comparison to previously reported results [which were all obtained in spectral codes, (16, 27)].

In contrast to earlier attempts to find UPOs by identifying “near recurrences” in time series and directly inputting them into a root-finder (8, 9, 16, 21), we instead conduct a search via gradient-based optimization of a scalar loss function—without any explicit initial condition selection. This loss function is just a scaled norm of Eq. **2**,

[3] $$\begin{array}{c} L:=\frac{\|T^{\alpha }f^{T}\left(u\right)-u\|}{\left\|u\right\|}, \end{array}$$

and depends on the initial condition, u, an unknown shift, α and period, T. Gradients with respect to all of these variables can be computed efficiently using the JAX library (35) and its extensions (36). Typically we deem that guesses for which we can reduce the loss to L≤0.015 are suitable for passing to the Newton solve—direct convergence with the optimizer is too slow and so it is used as an effective preconditioner on guesses for Newton.

At Re=40 we will also explicitly target certain periods, $T^{*}$, and attempt to find periodic orbits with average dissipation above some thresholds $D^{*}$ [such UPOs were largely missing from previous results, (16, 22, 27)]. We do this by adding appropriate terms to the loss,

[4a] $$\begin{array}{cc} L_{T} & :=\frac{\|T^{\alpha }f^{T}\left(u\right)-u\|}{\left\|u\right\|}+\gamma \;{\left(T-T^{*}\right)}^{2}, \end{array}$$

[4b] $$\begin{array}{cc} L_{D} & :=\frac{\|T^{\alpha }f^{T}\left(u\right)-u\|}{\left\|u\right\|}+\kappa \;\sigma \left(\frac{D^{*}-{\left\langle D\right\rangle }_{T}}{\delta }\right), \end{array}$$

where the hyperparameters $\gamma =10^{-2}$, κ=100 and $\delta =10^{-2}$; σ(∙) denotes a sigmoid function leading to very harsh penalization if the dissipation time average falls below the threshold $D^{*}$. The penalization on the target periods is relatively weak, with $|T-T^{*}|=O\left(1\right)$ resulting in a $O(10^{-2})$ contribution to the loss. When explicitly searching for high dissipation events we relax our threshold on Newton-worthy guesses to $L_{D}=0.05$. We use an AdaGrad (37) optimizer with initial learning rate η=0.35 throughout.

The primitive variable formulation of JAX-CFD (32) allows for a constant background vertical velocity $v_{0}$. The basic ‘Kolmogorov’ flow described above has $v_{0}=0$ but the problem in principle allows for any uniform cross-stream $v_{0}$ which once prescribed is constant in time. The addition of this effect will alter the periodic orbits—many of which can presumably be continued by homotopy back to $v_{0}=0$—and may also introduce new solutions in saddle node bifurcations that are not relevant to the original configuration. Here, we find the addition of this effect to be positive overall in that it prevents the optimizer from getting stuck in shallow local minima—which is a common feature when searching directly with the spectral, vorticity formulation of JAX-CFD. Similar observations have been made in a recent attempt to find periodic orbits in a variational formulation, where nonsolenoidal velocity fields were used as initial guesses (27). No constant background flow is possible in the spectral version of JAX-CFD which is used to perform Newton convergence on the UPOs after the gradient-based optimization, because we solve for the induced velocity via Δψ=−ω assuming a periodic streamfunction ψ, from which $u=\partial_{y}\psi$ and $v=-\partial_{x}\psi$.

For short periods (e.g., T≲10 at Re=40), the weak background vertical velocity only weakly effects the UPO and the spectral Newton solve is capable of converging the nearby $v_{0}=0$ solution in a few steps. For longer periods the weak vertical flow has more of an impact, but can often successfully be efficiently removed with an additional optimization run penalizing the vertical velocity,

[5] $$\begin{array}{c} L_{V}:=\frac{\|T^{\alpha }f^{T}\left(u\right)-u\|}{\left\|u\right\|}+\mu \;v_{0}^{2}, \end{array}$$

where we set $\mu =10^{3}$ and use a smaller learning rate (typically $\eta =10^{-2}$) to carefully deform the near-closed loop into one with near-zero vertical velocity.

## Unstable Periodic Orbits

### Density of States at *Re = 40*.

We first demonstrate the power of our approach in the more well-studied problem of Kolmogorov flow at Re=40. This configuration has been examined by a number of previous authors (16, 22, 24, 26, 27) though we still know very little about the density of states as a function of their period, ρ(T), and have not managed to identify any localized high dissipation UPOs. Motivated by this we conduct a sweep for solutions with specific periods using loss function [**4a**]. Due to the importance of “prime cycles” in periodic orbit theory (20), we first focus on short periods T∈[2,10]. We increment the target $T^{*}$ in values of 0.5 within this range and seed 50 optimization calculations at each $T^{*}$. Each calculation is initialized with a random snapshot from the turbulent attractor, an initial guess for the period $T^{0}=T^{*}$ and zero initial shift, $\alpha^{0}=0$. Previously four solutions were known to exist in this range, with periods T∈{2.83,2.92,5.38,6.72}.

We also initialize separate searches for high dissipation solutions using loss function [**4b**]. We perform three computations searching for solutions with average dissipation rates above threshold values $D^{*}\in \left\{0.12,0.15,0.2\right\}$. The computations are initialized in a similar way to those described previously, though with the starting snapshots constrained to have dissipation values $D>D^{*}$. We fix the initial period in this search at $T^{0}=4$ and the shift again at $\alpha^{0}=0$.

Large numbers of solutions are converged within the variable $v_{0}$ formulation, though success is not uniform across target $T^{*}$. For example, we find many solutions when $T^{*}\in \left\{5,5.5,6\right\}$, which when introduced into the $v_{0}=0$ spectral solver all collapse to the well-known UPO at T=5.38 (similar behavior is found close to T=2.83 and its integer multiples). Finding these common solutions is expected, but we do also find very large numbers of solutions which were previously unknown. Indeed, for T<8 we converge 38 unique solutions (detailed in *SI Appendix*), including many high dissipation states that have been inaccessible to previous search methods.

**Fig. 1.**
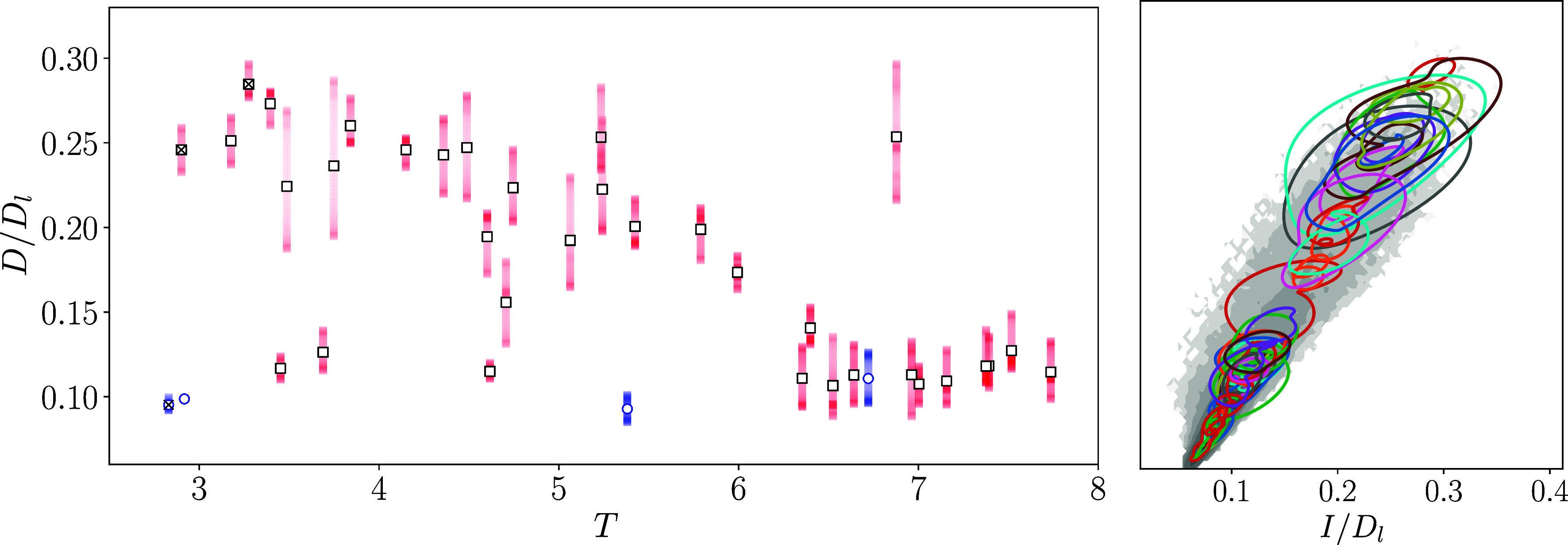
(*Left*) Dissipation against period of all converged orbits with periods T<8 at Re=40 (average dissipation rate is shown with a white square). The four previously known solutions listed in ref. 16 are identified in blue with mean dissipation indicated by a circle at T∈{2.83,2.92,5.38,6.72} (the solution at T=7.16 may have also been found in ref. 27). Solutions additionally identified with a cross are shown later in Fig. 2. (*Right*) Energy production versus dissipation for all converged solutions at Re=40, including some longer orbits not shown on the left (all converged UPOs are listed in *SI Appendix*). The probability density for the turbulent state (computed from a long calculation run for $t_{\text{long}}=2.5\times 10^{5}$) is shown in gray. Contour levels are spaced logarithmically with a minimum value of $10^{-6}$. All values are normalized by the laminar value $D_{l}=Re/\left(2n^{2}\right)$.

Remarkably, these short-period solutions appear to span nearly the full range of production and dissipation events in the overall flow (see the *Right* panel in Fig. 1). What is missing is actually the low dissipation events—these are associated with slower dynamics and the UPOs tend to have longer periods. To find these states, we also searched for solutions with target periods $T^{*}\in \left\{12.5,15,17.5,20\right\}$. Generally, the optimizer struggles for these longer orbits, as $f^{T}\left(u\right)$ is likely far away on the solution manifold when starting from random initial conditions and gradient information is not particularly helpful. However, we do obtain several longer solutions—most are listed in ref. 16—which are all low dissipation. This aspect of the work would benefit from a more considered approach to initial condition selection to ensure that $f^{T}\left(u\right)$ is “nearby” on the solution manifold without necessarily requiring a near recurrence.

**Fig. 2.**
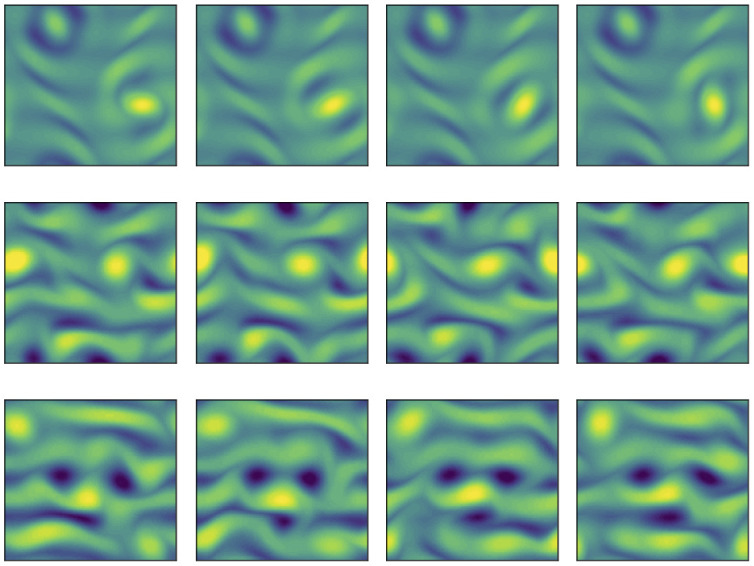
Out-of-plane vorticity are extracted at four points equispaced-in-time over three UPOs at Re=40. (*Top*) Shortest orbit at Re=40—the previously known T=2.83 solution with average dissipation rate $\langle D/D_{l}\rangle =0.095$. (*Middle*) A new high dissipation UPO with T=2.90 and average dissipation rate $\langle D/D_{l}\rangle =0.246$. (*Bottom*) A new high dissipation UPO with T=3.27 and average dissipation rate $\langle D/D_{l}\rangle =0.285$. In all cases, contours run for −10 to 10. Each of these solutions is identified in Fig. 1 with a cross.

We examine some of the Re=40 UPOs in Fig. 2, where we report snapshots of the out-of-plane vorticity at four points along the orbit. The low dissipation solutions (the T=2.83 UPO is shown in this figure) all share a common structure, with vortical structures sitting on top of a pair of slanted stripes of vorticity (which are reminiscent of the first nontrivial equilibrium solution in this configuration, see ref. 26). In the T=2.83 case the period corresponds to a complete rotation of an elliptical vortex patch located to the right of the panel; the orbit with T=5.38 is similar but involves the rotation of a like-signed vortex pair (see, e.g., ref. 16).

In contrast, the high dissipation structures display a much greater variety of dynamics. For instance, one of the two “bursting” UPOs reported in Fig. 2 features a crystal-like structure with four large-amplitude vortex cores that are maintained over the period. The other high dissipation solution in Fig. 2 features a strong dipole structure that makes its way through the domain from left to right. Unsurprisingly, many of the new solutions have higher-dimensional unstable manifolds than those that have been previously documented (see full details in *SI Appendix*).

### Periodic Orbits at *Re = 100*.

We now turn our attention to the strongly turbulent case at Re=100, where only a handful of solutions have been obtained by any previous method (16). The nine solutions obtained previously at this value of Re were all of short period, T<5. The majority of solutions we report here were computed using the basic loss function [**3**] without additional physics (e.g., as included in Eqs. 4a and 4b).

We initialize large numbers of computations with starting periods selected from $T^{0}\in \left\{1,2,3,4,5\right\}$, with additional calculations also performed $T^{0}=2.5$, motivated by the success rate of the $T^{0}=2$ and $T^{0}=3$ calculations. We stopped these computations once a batch of O(100) guesses failed to yield a new solution not already contained in our collection of converged UPOs. We also perform a high dissipation search using loss function [**4b**] for periodic orbits with average dissipations $\langle D/D_{l}\rangle \geq 0.03$, though find this to be much less effective than the equivalent computations at Re=40. In contrast, the standard loss function [**3**] returns a wide range of periodic orbits without augmentation, rather than the same subset of solutions. Overall, we observe a success rate for converging solutions of between 5 and 15%, depending on the choice of starting period. It is important to emphasize that the starting point in the process is a random snapshot from a turbulent calculation and should be contrasted to other methods with a success rate close to zero where guesses were more carefully constructed (e.g., the recurrent flow analysis in ref. 16 produced nine solutions from an analysis of $10^{5}$ time units of data).

**Fig. 3.**
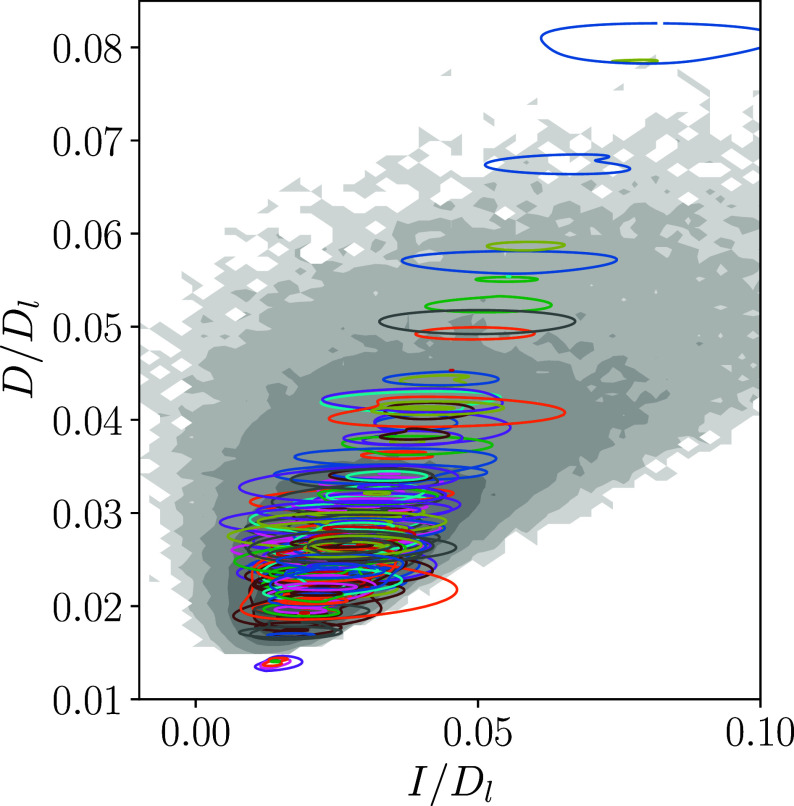
Energy production rate against dissipation rate at Re=100. Gray background is the PDF computed from a long turbulent computation with $t_{\text{long}}=2.5\times 10^{5}$. Contour levels are spaced logarithmically with a minimum value of $10^{-6}$. Closed loops are the two-dimensional projections of the 151 converged UPOs. All values are normalized by the laminar value $D_{l}=Re/\left(2n^{2}\right)$. All periodic orbits along with relevant properties (period, shift, Floquet exponents) are listed in *SI Appendix*.

Our computations have so far yielded 151 unique UPOs at Re=100, and the states are summarized in Fig. 3 (and listed in full in *SI Appendix*). Here, we see that most of the UPOs appear to be highly localized in phase space, nearly all appearing as small closed loops in the two-dimensional projection. The states appear even more localized when visualized in terms of their kinetic energy, E (*SI Appendix*, Fig. S4), and full coverage of the associated turbulent PDFs will require many more states than the equivalent computations at Re=40.

**Fig. 4.**
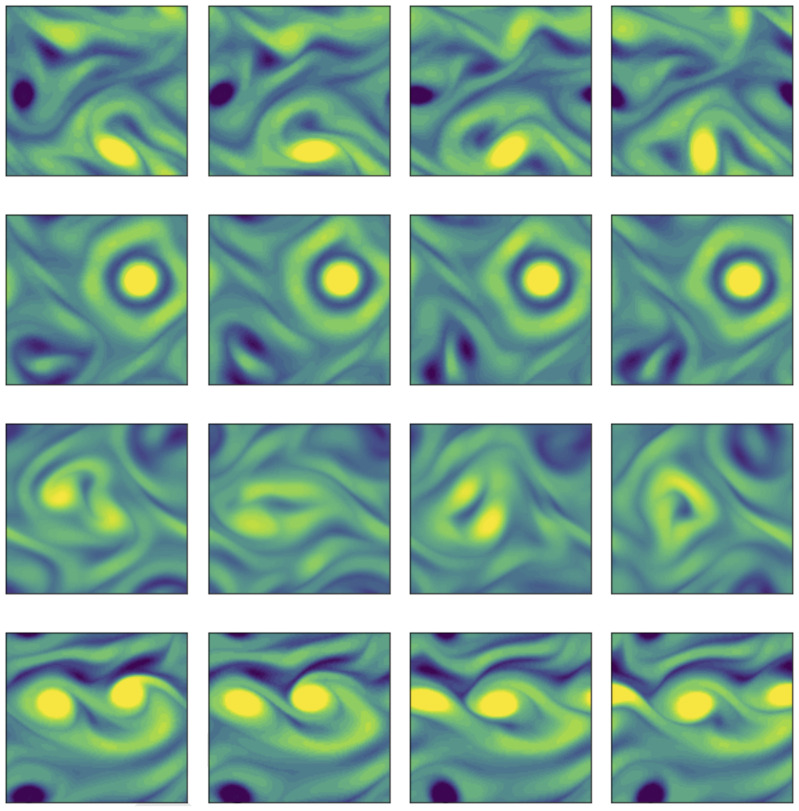
Out-of-plane vorticity are extracted at four points equispaced-in-time over four UPOs at Re=100. From top to bottom, the UPOs have the following periods and average dissipation rates: $\left(T,\left\langle D/D_{l}\right\rangle \right)=\left(1.424,0.057\right)$, (1.794,0.053), (4.212,0.027), and (1.164,0.078) (for full details of converged solutions see *SI Appendix*). Vorticity contour levels run between ±10.

We visualize some of the converged UPOs in Fig. 4. The solutions show a wide variety of different dynamical behaviors. Often, the states are dominated by two large vortex patches [expected due to the inverse cascade, (38)]—see middle two rows of Fig. 4. There is wide variation within this type of solution, in addition to the behavior in figure (e.g., where we see a strong stationary vortex and a corotating pair), there are also states with three or more like-signed corotating vortices—see further details in *SI Appendix*. The more strongly dissipative states (top and bottom rows of Fig. 4) show an even greater variety of vorticity dynamics, and further work will be required to assess the various “classes” of UPO discovered by our approach.

## Markov Chains from Periodic Orbit Shadowing

### Identifying UPO Shadowing.

Given the wide range of converged UPOs found, and the particularly good coverage of the *I*–*D* plane at Re=40, we now attempt to label snapshots of a turbulent time series by which UPO is “closest,” in an attempt to verify and visualize the original conjecture by Hopf (1). Our objective is to use our library of UPOs to partition the state space and convert turbulent orbits to realizations of a discrete-time Markov process.

To accurately measure distance to the nearest UPO, we have trained highly accurate deep convolutional autoencoders in a “DenseNet” (39, 40) configuration (see *Materials and Methods* for full architectural and training details) and will use an observable based on the latent representations in these networks, rather than using a distance between snapshots in physical space. The accuracy of these models has been demonstrated in our recent work (41) over a wide range of Re—accuracy is maintained even on the rare, highest dissipation events.

The autoencoders consist of an encoder, $E:R^{N_{x}\times N_{y}}\to R^{m}$ (here, m=128 at Re=40 and m=512 at Re=100), and decoder $D:R^{m}\to R^{N_{x}\times N_{y}}$ such that [D○E](ω)≈ω. Given an encoded snapshot, E(ω), we first construct a streamwise-shift invariant observable by projecting E onto so-called “latent Fourier modes,” which are eigenvectors of a discrete shift operator $T_{\alpha }E\left(\omega \right):=E\left(T^{\alpha }\omega \right)$ for some fixed streamwise shift α. We then use these projections to build a vector observable ψ(ω), which has the property $\psi \left(T^{s}\omega \right)=\psi \left(\omega \right)$∀s∈R (full details in *Materials and Methods*). Finally, we compute the period-averaged value of ψ for each periodic orbit as well as each of its 15 discrete-symmetry copies, $\left\{{\left\langle \psi \left(S^{m}R^{q}f^{t}\left(\omega_{j}\right)\right)\right\rangle }_{T}\;:\;0\leq m\leq 7,\;q\in \left\{0,1\right\}\right\}$.

The nearest periodic orbit to a snapshot ω is then determined according to

[6] $$\begin{array}{c} j^{*}={\arg\;\min}_{j}\;\;{\text{min}}_{m,q}{\left\|\psi \left(\omega \right)-{\left\langle \psi \left(S^{m}R^{q}f^{t}\left(\omega_{j}\right)\right)\right\rangle }_{T}\right\|}_{2}, \end{array}$$

where $1\leq j\leq N_{p}$ ($N_{p}$ is the total number of periodic orbits in our library at a given Re) and we search over the discrete symmetries at every time instant. A comparison to the time-average of the UPO embeddings is robust as the majority of our UPOs are short and localized in phase space, though more sophisticated methods could search over the time direction too.

We construct long trajectories of length $T=2.5\times 10^{4}$ at Re=40 and $T=10^{4}$ at Re=100, where snapshots are separated by δt=1 in the former case and δt=0.25 in the latter. Snapshot spacing in each is motivated by the typical period of the UPOs in our library (e.g., T∼5 is common at Re=40 while a plurality of solutions at Re=100 have 1≤T≤2) and the motivation to be able to observe “shadowing” of periodic solutions if such dynamics occurs. The turbulent time series are converted to sequences of labels of the form $\;{\text{PO}}_{i}\to \;{\text{PO}}_{i}\to \;{\text{PO}}_{i}\to \;{\text{PO}}_{k}\to \;{\text{PO}}_{j}\to \;{\text{PO}}_{j}\to \cdots$ (for example).

### Discrete-Time Markov Chains and Statistical Predictions.

**Fig. 5.**
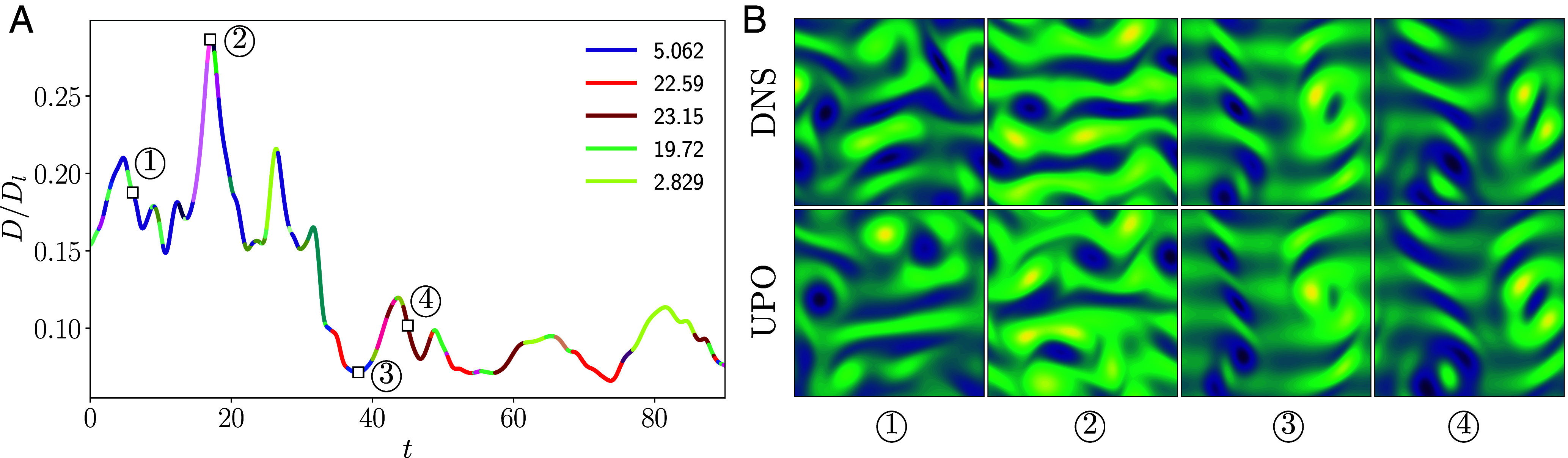
(*A*) Example time evolution of dissipation rate at Re=40, colored according to the nearest UPO as determined by Eq. **6**. The five most frequently visited UPOs in this interval are highlighted in the legend, labeled by their period, T. (*B*) Snapshots of vorticity (*Top*) from the direct numerical simulation (DNS) used to generate the dissipation plot at times identified with squares/numbers in (*A*), along with snapshots from the closest UPO (*Bottom*), where we have selected the horizontal shift, s, and the time along the orbit, τ, to minimize $\|\omega -T^{s}f^{\tau }\left(\omega_{\mathrm{PO}}\right){\|}_{2}$.

An example of the UPO-labeling protocol described above is reported in Fig. 5, where we have used a much finer δt for illustrative purposes. The example in the figure shows an extended high-dissipation bursting event which returns to more quiescent low-dissipation dynamics at around t∼30. The curve is colored according to which UPO is closest as determined by Eq. **6**, which indicates that there are extended periods of time where the flow remains close to a particular UPO, and that this particular sequence can be well described by just a small number of exact solutions. In fact, this example trajectory spends more than half its time in the vicinity of just four UPOs, with the high dissipation event repeatedly sampling the same solution (dark blue curves in Fig. 5, see also the figure legend). We compare snapshots along this example orbit with snapshots from the closest UPO in the right panel of the figure and can see many of the same qualitative flow features from the turbulence reproduced in the periodic solutions. It is also clear that the match could be improved—a more robust labeling protocol could also search over the time direction of all UPOs, albeit at significantly increased computational expense.

**Fig. 6.**
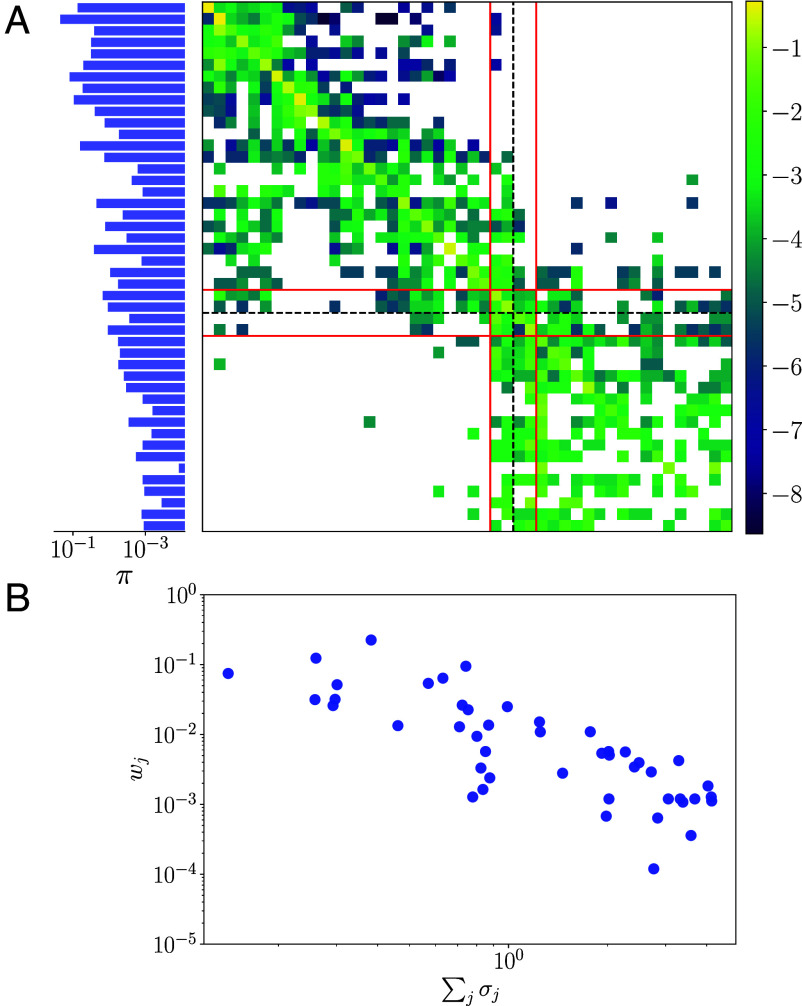
(*A*) Invariant measure π and transition matrix P at Re=40 (log of transition probabilities is shown, spacing between snapshots is δt=1). States are ordered from lowest to highest average dissipation rate (lowest at top/leftmost). The red horizontal lines identify the gateway, or “mixing” states discussed in the text. The red vertical lines are included to emphasize the wide range of states which have finite transition probabilities into this mixing region, encompassing both high- and low-dissipation events, while the dashed vertical line indicates the “threshold” value of $D/D_{l}=0.15$ discussed in the text. (*B*) The weights in the expansion [**7**]—which are also the invariant measure of the Markov chain $w_{j}=\pi_{j}$—plotted against the (real part of the) sum of growing Floquet exponents $\sum_{j}\sigma_{j}$, $\sigma_{j}>0$, for each UPO.

We now use the long time series described at the end of the previous section to construct transition probability matrices P, with elements $P_{\mathrm{ij}}:=P\left(\;{\text{PO}}_{i}\to \;{\text{PO}}_{j}\right)$. This quantity is trivially computed by simple counting of the transitions between states before normalizing rows $\sum_{j}P_{\mathrm{ij}}=1\;\forall i$. The transition matrix at Re=40 is reported in Fig. 6*A*, where the states have been ordered by dissipation (low dissipation top rows, highest at the bottom). We also show the invariant measure obtained via $\pi^{T}P=\pi^{T}$.

There are a number of interesting features present in the transition matrix which merit further discussion. The transition matrix generally shows the largest probabilities along its diagonal, which means that for most states the most likely outcome is to remain in the vicinity of that particular UPO for another time instant (δt=1 here at Re=40). This is consistent with a turbulent orbit shadowing individual recurrent solutions. The off-diagonal nonzero probabilities also show that transitions tend to occur between states with similar dissipation rates. The chaotic dynamics at Re=40 can be delineated into a low-dissipation “quiescent” regime and rarer, high-dissipation bursting events (roughly with normalized dissipation $D/D_{l}≳0.15$, see refs. 16, 24, and 26), and this delineation is clear in the transition matrix of Fig. 6 where states in the figure are ordered according to their average dissipation, and the value $D/D_{l}=0.15$ is identified with a dashed black line; there are a multitude of routes between the high dissipation states, though transitions to these bursting events appear to occur via a small number ∼4 of gateway, lower-dissipation UPOs which can also throw the system back to very low dissipations (see red lines in Fig. 6).

The Markovian view of turbulence can be extended to make statistical predictions in the spirit of periodic orbit theory (20, 29, 30). To do this we seek a fixed set of weights, ${\left\{w_{j}\right\}}_{j=1}^{N_{p}}$, where $N_{p}$ is the total number of UPOs found, such that the spatial (over the state space M) average of any observable $\gamma :M\to R^{n}$, can be constructed as a linear superposition of the statistics of the UPOs,

[7] $$\begin{array}{c} \Gamma \left(w\right)=\sum_{j=1}^{N_{p}}w_{j}\Gamma_{j}, \end{array}$$

where Γ is the average under consideration and $\Gamma_{j}$ is the same statistic computed for periodic orbit j. From our transition matrix, we are able to define the weights simply as $w_{j}\equiv \pi_{j}$, where $\sum_{j}w_{j}=1$ by definition. These weights are examined as a function of the instability of the underlying UPOs in the lower panel *B* of Fig. 6 (as measured by the sum of the growth rates from Floquet exponents). The weights are anticorrelated with the level of instability, where the highly unstable states are much less important in the reconstruction. This reflects the fact that the highly dissipative UPOs (which contribute to the very weak right tail of the distribution) tend to be much more unstable.

**Fig. 7.**
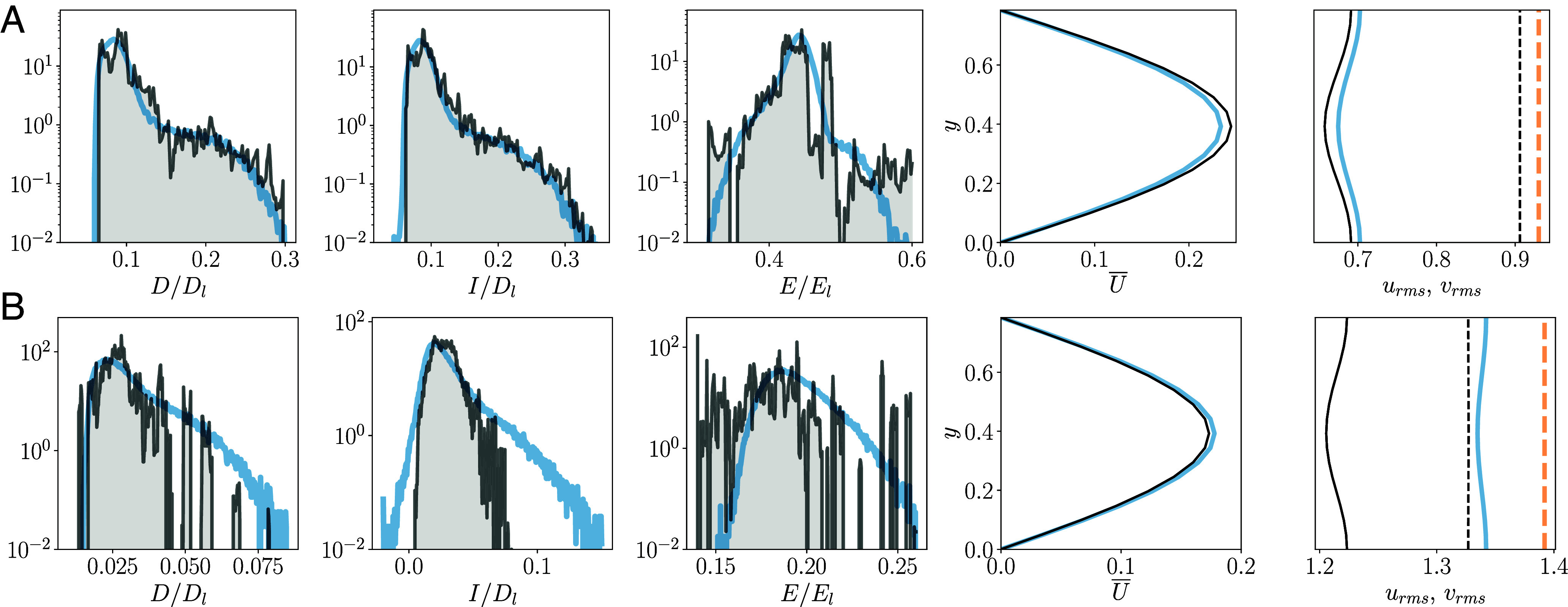
UPO-based predictions of statistics computed from the invariant measure of the Markov chain, which defines the weights in the UPO expansion (Eq. **7**), for (*A*) Re=40 and (*B*) Re=100 (transition matrix reported in *SI Appendix*). First three panels from left to right show PDFs of dissipation rate, production rate, and energy: the dashed blue lines are “ground truth” PDFs obtained from a long turbulent calculation ($t_{\text{long}}=2.5\times 10^{5}$) and the filled gray curves are the UPO reconstructions. The final two panels compare the mean velocity profile $\bar{U}\left(y\right)$ and the RMS velocity fluctuations (u and v are left and right in the panel)—averaged over the streamwise direction, discrete symmetries, and time. Blue and orange curves are the DNS ground truth for u and v, respectively, black curves the UPO reconstruction.

To demonstrate the performance of the above UPO expansion [**7**], Fig. 7*A* reports PDFs of the dissipation rate, energy production rate, and kinetic energy computed over a long time horizon ($t_{\text{long}}=2.5\times 10^{5}$) at Re=40 overlaid with UPO-based PDFs computed via expressions like Eq. **7**. The reproduction of both dissipation and production is remarkably complete—although we are missing some of the very lowest values associated with the longer orbits (as discussed previously). Most notable in both cases is the accurate reconstruction of the high-D/high-I tails, which has eluded all previous attempts (16, 42). The kinetic energy, E, is also reproduced to a fairly high standard [in particular compared to past attempts to apply periodic orbit theory, (16)]. The missing states at $E/E_{l}∼0.35$ and $E/E_{l}∼0.45$ can be plausibly linked to the missing lowest dissipation orbits. This is explored further in *SI Appendix* where we also show our solutions in the energy-dissipation plane.

The UPO predictions (again with the same weights) for the mean velocity profile and RMS velocity fluctuations are also tested in Fig. 7*A* (averaged over discrete symmetries as well as the streamwise coordinate and time, see refs. 16 and 24). All three are close to the true time-averaged values (e.g., errors O(1−2%) in $u_{\mathrm{rms}}$ and $v_{\mathrm{rms}}$), which again is a dramatic improvement in UPO-based prediction over the past state-of-the-art.

A similar analysis is performed using our large collection of 151 UPOs at Re=100 in Fig. 7*B*. The corresponding transition matrix is included in *SI Appendix* and again indicates shadowing but with likely transitions widely separated in average dissipation—though clearly the picture is incomplete because we are missing many important states as implied by our earlier results (e.g., Fig. 3). Nonetheless, the statistics reported in Fig. 7*B* are promising and represent a significant advance on what has been possible previously even at much lower Re. In particular, the mean profile is produced near perfectly, while the errors on the RMS velocities are only O(10%). The PDFs indicate that the missing states are associated with larger dissipation and energy values, and the UPO-search procedure outlined here provides a clear strategy for filling these gaps with further computation.

## Discussion

In this paper, we have assembled compelling evidence to support the viewpoint (often attributed to Hopf, see discussion in ref. 3) of turbulence as a high-dimensional pinball bouncing between unstable simple invariant sets. To do this we both i) designed a methodology for finding large numbers of dynamically relevant UPOs—a long-standing limitation in the field—and ii) presented an approach to accurately label turbulent snapshots by the closest UPO, where distance is measured in the latent space of a deep convolutional autoencoder. The result is a Markovian picture of turbulence, which yields at modest Re both insight into dynamical pathways and routes to extreme events along with robust statistical predictions for the chaotic dynamics.

The UPO search strategy is formed as a gradient-based optimization problem and is implemented in a fully differentiable flow solver. The loss-based approach allows for a targeted search for solutions with specific features (e.g., high dissipation, high energy) and application of the method at modest Re=40 revealed very large numbers of solutions with short periods—both high- and low-dissipation—that had been previously undetectable. The approach remains effective at the much higher value of Re=100, where we again found very large numbers of short solutions. The states at high Re appear to be highly localized in state space, and display a wealth of interesting vortical dynamics.

To label vorticity snapshots according to the closest UPO, we trained highly accurate deep convolutional autoencoders and measured similarity using an observable in the low-dimensional latent space of these networks. We were then able to treat long turbulent time series as Markov chains with each UPO being a distinct state, building transition matrices, and then using their invariant measure to make statistical predictions. The approach was particularly effective at Re=40, where the new library of UPOs covers nearly the full range of production and dissipation events seen by the fully turbulent state, and as a result, statistical predictions are robust. Even with an incomplete set of states at Re=100, the statistical predictions of the velocity moments are fairly robust and a substantial improvement on what has been possible even at much lower Re using earlier methods.

Despite the enormous numbers of new solutions converged, there are still important missing states at Re=40, and a large number of missing solutions at Re=100. Some of these gaps (e.g., the lower dissipation events at Re=40) could potentially benefit from an improved process of selecting the snapshots which are input to the optimizer. In the present configuration, improvements could involve something as simple as only selecting snapshots which return to a similar region of state space—not a near recurrence but a weaker requirement e.g. determined by the autoencoders trained here—or by searching over discrete symmetries. This is likely an important consideration when studying more complex three-dimensional flows.

All this being said, the wide gaps in coverage of the state space at Re=100, combined with the increasing computational cost to assemble the solutions, naturally raises some concerns about the applicability of the “UPO-pinball” concept to decompose statistics of higher-Re flows both in two dimensions and in three-dimensional, multiscale turbulence. For instance, converging a new solution here with T≈2.5 at Re=100 with $N_{x}\times N_{y}=512\times 512$ gridpoints requires on average ∼24 h of GPU compute on an Nvidia V100 card (this calculation assumes a 5% success rate starting from random turbulent snapshots). There is also the cost of training the autoencoders used to label snapshots (∼48 h of GPU time on a training dataset of $∼10^{5}$ snapshots) and the need for a long reference trajectory to construct a robust Markov chain—which itself yields accurate statistics that the UPOs are used to reconstruct.

Even if complete statistical coverage is challenging, the ability to search for exact recurrent flows with particular physical properties is exceptionally useful if the goal is to understand the importance of individual dynamical events within well-known statistical phenomena like the energy cascade. It is also worth noting that the three-dimensional case may differ substantially from the two-dimensional flows studied here: in 2D we expect that some of the solutions we obtain at higher Re may connect to solutions of the Euler equations as Re→∞ (41, 43), which are expected to take the form of domain-filling vortices, while wall-bounded turbulence is expected to require a more complex multiscale description with eddies of varying sizes attached to the boundaries (44). Early studies of weak, transient turbulence in 3D minimal flow units have shown that the statistics of individual UPOs can sometimes closely resemble the turbulence (8) itself which provides cautious optimism for the 3D case.

The UPO search approach outlined in this paper allows us to explicitly target the gaps in the PDFs we have constructed so far, and we believe that this ability means that a robust statistical coverage with UPOs is possible though at present the computation time required does appear extreme. There is scope to improve many aspects of the methods that would potentially speed up the search, including i) a more considered initial snapshot selection and ii) modifying the optimization (e.g., via change of norm) to improve convergence rates. The promise of such a representation extends far beyond simple statistical reconstruction: the UPO representation allows for computation of sensitivities of a chaotic system where gradients of statistics are usually not computable, while the grand hope would be to use a construction at one Re to assess the role of individual dynamical events in the statistics at much higher Re via continuation of the solutions and their weights.

## Materials and Methods

### Simulations.

Simulations with the finite-difference version of JAX-CFD were performed on grids of size $N_{x}\times N_{y}=256\times 256$ at Re=40 and $N_{x}\times N_{y}=512$ at Re=100. For the Newton-solve component of our algorithm, we use the spectral version of JAX-CFD (34), and we matched the resolution in the spectral simulations to those in the precursor finite difference optimization. The timestep was determined by a CFL condition based on a velocity estimate which is twice that of the laminar base profile, $2\times Re/(2n^{2})$, which is typically much larger than velocities observed in the turbulent regime.

The spectral version of the code solves the Navier–Stokes equations in vorticity-velocity form,

[8] ∂tω+u·∇ω=1ReΔω−ncos(ny),

with $\omega :=\left(\nabla \times u\right)\cdot \hat{z}$ (compare to Eq. 1). Unlike the finite-difference, primitive variable formulation, no background constant flow is possible. The velocity field at each timestep is that induced by the vorticity, and is found from the solution of a Poisson equation, Δψ=−ω, where the streamfunction ψ yields the induced velocity components via $u=\partial_{y}\psi$, $v=-\partial_{x}\psi$.

### Neural Network and Distance Metric.

#### Architecture.

The convolutional autoencoders are a combination of an encoder, $E_{m}$, and decoder, $D_{m}$, such that

[9] $$\begin{array}{c} A_{m}\left(\omega \right):=\left[D_{m}○E_{m}\right]\left(\omega \right)\approx \omega . \end{array}$$

Dimensionality reduction is performed with the encoder $E_{m}:R^{N_{x}\times N_{y}}\to R^{m}$, where we fix m=128 for the Re=40 data and m=512 at Re=100. Performance at a variety of m for various Re is examined in ref. 41.

The encoder is a fully convolutional architecture, with dimensionality reduction done by max pooling after a “dense block” of convolutions (39, 40). Each dense block consists of three successive convolutional layers, with each receiving the output of the previous convolution concatenated with outputs of all other upstream layers within the same dense block. Each convolution within a dense block creates an additional 32 feature maps. After each dense block, we apply max pooling followed by a single convolutional layer to reduce the number of feature maps to 32, and the full encoder is made up of six dense-block/max pooling combinations. At the innermost representation, the encoder produces an image of shape 4×8×M, where M=m/32 (m is the specified dimensionality of the innermost latent representation). As described in ref. 41, the vertical value of “8” is set by the discrete shift reflect symmetry in the Kolmogorov flow.

Throughout the network, we use “GELU” activation functions (45), apart from the decoder output where tanh is used (input data is normalized $\omega \to \omega /\omega_{\text{norm}}$ such that max|ω(x,y)|≤1). The decoder module is similar in structure to the encoder but in reverse, with upsampling applied in place of max pooling. Further details can be found in ref. 41 while the architecture and weights are available in ref. 46.

#### Training.

We use a loss function which is a modified “mean square error”:

[10] $$\begin{array}{c} L_{\mathrm{AE}}:=\frac{1}{2N}\sum_{j=1}^{N}\|A_{m}\left(\omega_{j}\right)-\omega_{j}{\|}^{2}+\frac{1}{2N}\sum_{j=1}^{N}{\left\|A_{m}^{2}\left(\omega_{j}\right)-\omega_{j}^{2}\right\|}^{2}, \end{array}$$

where the additional term (a mean square error on the vorticity squared) is designed to encourage the network to learn an effective representation of the—much rarer—high dissipation events (rather than changing the distribution the underlying data is drawn from) where this term is an increasingly important contribution to the overall loss.

We train on $N=10^{5}$ samples generated at each Re, consisting of 1,000 independent trajectories with snapshots spaced by an advective time unit. We apply data augmentation: randomly shifting in x and y, as well as applying the rotational symmetry. An Adam optimizer (47) is used for all models, with learning rate $\eta =5\times 10^{-4}$, and we train for 500 epochs with a batch size of 64. The performance of these models is examined in detail in ref. 41 and the reader is referred there for further detail.

#### Latent Fourier analysis.

Latent Fourier analysis is an interpretability technique for autoencoders originally described in ref. 26 which exploits the continuous symmetry in the governing equations/boundary conditions. In latent Fourier analysis, we seek an operator to perform continuous shifts for embeddings of vorticity fields, i.e.,

[11] $$\begin{array}{c} T_{\alpha }E_{m}\left(\omega \right):=E\left(T^{\alpha }\omega \right), \end{array}$$

where we pick α=2π/n with n∈N, such that $T_{\alpha }^{n}E\left(\omega \right)=E\left(\omega \right)$. The eigenvalues of $T_{\alpha }$ are then the nth roots of unity, $\lambda_{j}=\exp\left(2\pi il_{j}/n\right)$, and we refer to $l_{j}\in Z$ as a latent wavenumber (26, 41).

In practice, we determine an approximate ${\hat{T}}_{\alpha }$ using the “dynamic mode decomposition” algorithm of ref. 48, which gives us the best (in a least squares sense) operator that maps between the test dataset of embeddings and their α-shifted counterparts. Empirically we observe a small number of nonzero latent wavenumbers which saturate as α is incrementally reduced ($l_{\max}=3$ at Re=40 and $l_{\max}=7$ at Re=100) which indicates that the networks embed patterns with a fundamental periodicity set by l. Each latent wavenumber is then (potentially highly) degenerate.

We can then write down a representation of the embedding of a snapshot subject to an arbitrary shift s∈R:

[12] $$\begin{array}{c} E_{m}\left(T^{s}\omega \right)=\sum_{l}P^{l}\left(E_{m}\left(\omega \right)\right)e^{\mathrm{ils}}, \end{array}$$

which makes a clear connection to a standard Fourier transform. Here, $P^{l}$ is the projector onto the degenerate eigenspace associated with wavenumber l:

$$\begin{array}{c} P^{l}\left(E_{m}\left(\omega \right)\right)=\sum_{k=1}^{d(l)}P_{k}^{l}\left(E_{m}\left(\omega \right)\right), \end{array}$$

and the projectors onto each of the d(l) degenerate directions are defined as $P_{k}^{l}\left(E_{m}\left(\omega \right)\right):=\left[{\left(\xi_{k}^{(l)†}\right)}^{H}E_{m}\left(\omega \right)\right]\xi_{k}^{\left(l\right)}$, where the $\xi_{k}^{\left(l\right)}$, where the $\xi_{k}^{\left(l\right)}$ are the eigenvectors in subspace l, while the dagger indicates the adjoint eigenvectors.

To define our shift invariant observable ψ(ω) we perform an SVD of projections onto individual eigenspaces associated with l≤3 (the analysis of ref. 41 indicates that the majority of energy is contained in these patterns), determining the degeneracy d(l) by the number of eigenvalues |λ|>0.9. The shift invariant observable is then determined using the left singular vectors from the SVD within each subspace, ${\left\{u_{l}^{k}\right\}}_{k=1}^{d(l)}$:

[13] $$\begin{array}{c} \psi \left(\omega \right):=\left(\begin{array}{c} {\left(u_{l=0}^{1}\right)}^{H}P^{0}\left(E\left(\omega \right)\right)\\ {\left(u_{0}^{2}\right)}^{H}P^{0}\left(E\left(\omega \right)\right)\\ \vdots \\ |{\left(u_{1}^{1}\right)}^{H}P^{1}\left(E\left(\omega \right)\right)|\\ \vdots \\ |{\left(u_{3}^{d(3)}\right)}^{H}P^{3}\left(E\left(\omega \right)\right)| \end{array}\right). \end{array}$$

Taking the absolute value of the projections onto l>0 ensures that $\psi \left(\omega \right)\approx \psi \left(T^{s}\omega \right)\;\forall s\in R$.

The utility and relevance of this observable to features in the flow, as compared to, e.g., measuring distances between vorticity fields in physical space, was established in ref. 41, where it has been shown i) that individual projections onto latent Fourier modes can be decoded (using the decoder $D_{m}$) into physically meaningful patterns resembling known simple invariant solutions and ii) measuring similarity between snapshots using ψ over ω as the observable significantly improves recurrent flow analysis for UPO detection.

## Supplementary Material

Appendix 01 (PDF)

## Data Availability

The code to search for and converge unstable periodic orbits in Kolmogorov flow with JAX-CFD are available in a GitHub repository (https://github.com/JacobSRPage/upo-ad). Additional data, including weights for the neural networks used to identify shadowing have been made available via the University of Edinburgh’s DataShare service (49).

## References

[1] E. Hopf, A mathematical example displaying features of turbulence. Commun. Pure Appl. Math. **1**, 303–322 (1948).

[2] D. W. Moore, E. A. Spiegel, A thermally excited non-linear oscillator. Astrophys. J. **143**, 871 (1966).

[3] F. Christiansen, P. Cvitanovic, V. Putkaradze, Spatiotemporal chaos in terms of unstable recurrent patterns. Nonlinearity **10**, 55–70 (1997).

[4] G. Kawahara, M. Uhlmann, L. van Veen, The significance of simple invariant solutions in turbulent flows. Annu. Rev. Fluid Mech. **44**, 203–225 (2012).

[5] M. D. Graham, D. Floryan, Exact coherent states and the nonlinear dynamics of wall-bounded turbulent flows. Annu. Rev. Fluid Mech. **53**, 227–253 (2021).

[6] R. R. Kerswell, Recent progress in understanding the transition to turbulence in a pipe. Nonlinearity **18**, R17–R44 (2005).

[7] B. Eckhardt, T. M. Schneider, B. Hof, J. Westerweel, Turbulence transition in pipe flow. Annu. Rev. Fluid Mech. **39**, 447–468 (2007).

[8] G. Kawahara, S. Kida, Periodic motion embedded in plane Couette turbulence: Regeneration cycle and burst. J. Fluid Mech. **449**, 291–300 (2001).

[9] P. Cvitanovic, J. F. Gibson, Geometry of the turbulence in wall-bounded shear flows: Periodic orbits. Phys. Scr. T142, 014007 (2010).

[10] J. M. Hamilton, J. Kim, F. Waleffe, Regeneration mechanisms of near-wall turbulence structures. J. Fluid Mech. **287**, 317–348 (1995).

[11] F. Waleffe, On a self-sustaining process in shear flows. Phys. Fluids **9**, 883–900 (1997).

[12] P. Hall, S. Sherwin, Streamwise vortices in shear flows: Harbingers of transition and the skeleton of coherent structures. J. Fluid Mech. **661**, 178–205 (2010).

[13] B. Eckhardt, H. Faisst, A. Schmiegel, J. Schumacher, “Turbulence transition in shear flows” in Advances in Turbulence IX: Proceedings 9th European Turbulence Conference, Southampton, I. P. Castro , Eds. (CISME, 2002), p. 701.

[14] R. R. Kerswell, O. R. Tutty, Recurrence of travelling waves in transitional pipe flow. J. Fluid Mech. **584**, 69–102 (2007).

[15] J. F. Gibson, J. Halcrow, P. Cvitanovic, Visualizing the geometry of state space in plane Couette flow. J. Fluid Mech. **611**, 107–130 (2008).

[16] G. J. Chandler, R. R. Kerswell, Invariant recurrent solutions embedded in a turbulent two-dimensional Kolmogorov flow. J. Fluid Mech. **722**, 554–595 (2013).

[17] B. Suri, L. Kageorge, R. O. Grigoriev, M. F. Schatz, Capturing turbulent dynamics and statistics in experiments with unstable periodic orbits. Phys. Rev. Lett. **125**, 064501 (2020). 32845663 10.1103/PhysRevLett.125.064501

[18] G. Yalniz, B. Hof, N. B. Budanur, Coarse graining the state space of a turbulent flow using periodic orbits. Phys. Rev. Lett. **126**, 244502 (2021). 34213943 10.1103/PhysRevLett.126.244502

[19] M. C. Krygier, J. L. Pughe-Sanford, R. O. Grigoriev, Exact coherent structures and shadowing in turbulent Taylor–Couette flow. J. Fluid Mech. **923**, A7 (2021).

[20] P. Cvitanović, R. Artuso, R. Mainieri, G. Tanner, G. Vattay, Chaos: Classical and Quantum (Niels Bohr Institute, Copenhagen, Denmark, 2016).

[21] D. Viswanath, Recurrent motions within plane Couette turbulence. J. Fluid Mech. **580**, 339–358 (2007).

[22] D. Lucas, R. R. Kerswell, Recurrent flow analysis in spatiotemporally chaotic 2-dimensional Kolmogorov flow. Phys. Fluids **27**, 045106 (2015).

[23] Y. H. Lan, P. Cvitanovic, Variational method for finding periodic orbits in a general flow. Phys. Rev. E **69**, 016217 (2004). 10.1103/PhysRevE.69.016217 14995703

[24] M. Farazmand, An adjoint-based approach for finding invariant solutions of Navier–Stokes equations. J. Fluid Mech. **795**, 278–312 (2016).

[25] J. Page, R. R. Kerswell, Searching turbulence for periodic orbits with dynamic mode decomposition. J. Fluid Mech. **886**, A28 (2020).

[26] J. Page, M. P. Brenner, R. R. Kerswell, Revealing the state space of turbulence using machine learning. Phys. Rev. Fluids **6**, 034402 (2021).

[27] J. Parker, T. Schneider, Variational methods for finding periodic orbits in the incompressible Navier–Stokes equations. J. Fluid Mech. **941**, A17 (2022).

[28] D. Lucas, T. Yasuda, Stabilization of exact coherent structures in two-dimensional turbulence using time-delayed feedback. Phys. Rev. Fluids **7**, 014401 (2022).

[29] R. Artuso, E. Aurell, P. Cvitanovic, Recycling of strange sets: I. Cycle expansions. Nonlinearity **3**, 325–359 (1990).

[30] R. Artuso, E. Aurell, P. Cvitanovic, Recycling of strange sets: II. Applications. Nonlinearity **3**, 361–386 (1990).

[31] P. Cvitanovic, Periodic orbits as the skeleton of classical and quantum chaos. Physica D **51**, 138–151 (1991).

[32] D. Kochkov , Machine learning–accelerated computational fluid dynamics. Proc. Natl. Acad. Sci. U.S.A. **118** (2021). 10.1073/pnas.2101784118 PMC8166023 34006645

[33] N. Platt, L. Sirovich, N. Fitzmaurice, An investigation of chaotic Kolmogorov flows. Phys. Fluids **3**, 681–696 (1991).

[34] G. Dresdner , Learning to correct spectral methods for simulating turbulent flows. arXiv [Preprint] (2022). 10.48550/arXiv.2207.00556 (Accessed 1 August 2023).

[35] J. Bradbury , JAX: composable transformations of Python+NumPy programs. Github. http://github.com/google/jax. Accessed 1 August 2022.

[36] P. Kidger, C. Garcia, “Equinox: Neural networks in JAX via callable PyTrees and filtered transformations” in *Differentiable Programming workshop at Neural Information Processing Systems, 2021* (2021).

[37] J. Duchi, E. Hazan, Y. Singer, Adaptive subgradient methods for online learning and stochastic optimization. J. Mach. Learn. Res. **12**, 2121–2159 (2011).

[38] G. Boffetta, R. E. Ecke, Two-dimensional turbulence. Annu. Rev. Fluid Mech. **44**, 427–451 (2012).

[39] G. Huang, Z. Liu, L. van der Maaten, K. Q. Weinberger, “Densely connected convolutional networks” in *Proceedings of the IEEE Conference on Computer Vision and Pattern Recognition* (2017).

[40] G. Huang, Z. Liu, G. Pleiss, L. Van Der Maaten, K. Weinberger, Convolutional networks with dense connectivity. IEEE Trans. Pattern Anal. Mach. Intell. **44**, 8704–8716 (2019). 10.1109/TPAMI.2019.2918284 31135351

[41] J. Page, J. Holey, M. P. Brenner, R. R. Kerswell, Exact coherent structures in two-dimensional turbulence identified with convolutional autoencoders. arXiv [Preprint] (2023). 10.48550/arXiv.2309.12754 (Accessed 15 October 2023).

[42] D. Lucas, R. Kerswell, Spatiotemporal dynamics in two-dimensional Kolmogorov flow over large domains. J. Fluid Mech. **750**, 518–554 (2014).

[43] D. Zhigunov, R. O. Grigoriev, Exact coherent structures in fully developed two-dimensional turbulence. J. Fluid Mech. **970**, A18 (2023).

[44] Q. Yang, A. P. Willis, Y. Hwang, Exact coherent states of attached eddies in channel flow. J. Fluid Mech. **862**, 1029–1059 (2019).

[45] D. Hendrycks, K. Gimpel, Gaussian error linear units (GELUs). arXiv [Preprint] (2016). 10.48550/arXiv.1606.08415 (Accessed 10 November 2023).

[46] J. Page, P. Norgaard, M. P. Brenner, R. R. Kerswell, “Neural network weights related to ‘Recurrent patterns as a basis for two-dimensional turbulence: predicting statistics from structures”’. DataShare. 10.7488/ds/7732. Deposited 7 May 2024. PMC11161751 38820003

[47] D. P. Kingma, J. Ba, “Adam: A method for stochastic optimization” in 3rd International Conference on Learning Representations, ICLR 2015, San Diego, CA, May 7–9, 2015, Conference Track Proceedings, Y. Bengio, Y. LeCun, Eds. (2015).

[48] P. J. Schmid, Dynamic mode decomposition of numerical and experimental data. J. Fluid Mech. **656**, 5–28 (2010).

[49] J. Page, P. Norgaard, M. P. Brenner, R. R. Kerswell, Recurrent flow patterns as a basis for two-dimensional turbulence: Predicting statistics from structures. UPO-AD. https://github.com/JacobSRPage/upo-ad. Deposited 6 November 2023. 10.1073/pnas.2320007121 PMC11161751 38820003

