# Supplementary material for: Recurrent flow patterns as a basis for two-dimensional turbulence: Predicting statistics from structures
Source: Proc Natl Acad Sci U S A. 2024 May 31;121(23):e2320007121. doi: 10.1073/pnas.2320007121 (PMC11161751; doi:10.1073/pnas.2320007121)
Supplement: Supplementary file 1 — Appendix 01 (PDF) [file pnas.2320007121.sapp.pdf]

Supporting Information for

Recurrent flow patterns as a basis for two-dimensional turbulence: predicting statistics from structures

Jacob Page, Peter Norgaard, Michael P. Brenner & Rich R. Kerswell

Jacob Page
E-mail: jacob.page@ed.ac.uk

This PDF file includes:

Supporting text
Figs. S1 to S4
Tables S1 to S2
SI References

Supporting Information Text

Latent distance to UPOs

As described in the main text, the distance to periodic orbit j is determined according to

$$d_{\psi}^{j}(\omega) := \min_{m,q} \|\boldsymbol{\psi}(\omega) - \langle \boldsymbol{\psi}(\mathscr{S}^m \mathscr{R}^q f^t(\omega_j))\rangle_T\|_2, \qquad [1]$$

with the closest UPO following from $j^* = \arg\min_j d_{\psi}^{j}(\omega)$. In figure S1 we report the distance (1) to all UPOs found at $Re = 40$ for a short turbulent trajectory (the same one considered in figure 5 in the main text), along with the time evolution of the dissipation rate. We highlight two particular solutions which are frequently visited in this turbulent episode, a high dissipation solution (blue) which plays a dominant role in the burst, and a common low dissipation state (orange) which is shadowed for an extensive period around $t \sim 80$.

Further results at $Re = 100$

The higher $Re = 100$ transition matrix is included here in figure S2 (compare to the $Re = 40$ results in figure 6 of the main paper), along with the invariant measure used to compute the statistics in figure 7 of the manuscript. There is no clear distinction between low/high dissipation states and transitions can apparently occur between widely separated UPOs (in terms of dissipation). However, these results are clearly likely to be impacted significantly by the large number of missing states – e.g. see the $I - D$ plot in figure 3 of the main paper, and a clearer picture will likely emerge as we continue to converge new solutions in future calculations. The relationship between the weights defined by the invariant measure of the $Re = 100$ transition matrix and the unstable growth rates of the associated UPOs are also reported in figure S2. Unlike the $Re = 40$ results, it is challenging to identify a clear relationship, which again may be resolved after computation of more solutions.

In addition to the transition matrix, we also report snapshots of spanwise vorticity for five further UPOs at $Re = 100$ in figure S3 to demonstrate the wealth of vorticity dynamics contained in the UPO library. These include localised co-rotating three-vortex states, as well as large quiescent vortex patches.

Periodic orbit details

Here we report details of the UPOs we have found via automatic differentiation at both $Re = 40$ and $Re = 100$ in tables S1 and S2 respectively, including their leading Floquet exponent and the dimension of the unstable manifold.

In addition, we also include a visualisation of the UPOs at both $Re = 40$ and $Re = 100$ in the energy-dissipation plane (figure S4) to better illustrate the difficulty in reconstruction of the PDF of E (particularly at $Re = 40$ – see figure 7a in the main paper) compared to the dissipation rate. It is clear from the figure that at $Re = 40$ we are missing low-energy, low-dissipation states in our solution library, which can be reasonably attributed to either (i) our focus on searching for short orbits or (ii) our lack of a search over discrete symmetries for pre-periodic orbits, which we hope to address in future work.

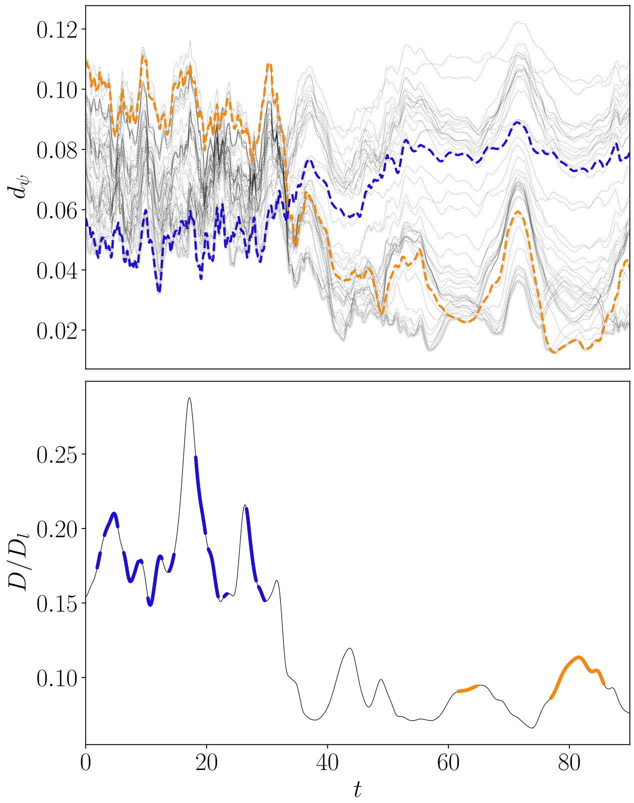

Fig. S1. (Top) Distance $d_\psi(f^t(\omega))$ to *all* UPOs along a short turbulent trajectory at $Re = 40$ (grey lines). The values shown have been normalised by $\|\langle \boldsymbol{\psi}(f^t(\omega))\rangle\|$, where $\langle\bullet\rangle$ indicates and average over the long 2.5×10^4 trajectory described in the main text. Two frequently visited UPOs are highlighted in orange ($T = 2.829$, low dissipation $\langle D/D_l\rangle_T = 0.095$) and blue ($T = 5.062$, high dissipation $\langle D/D_l\rangle_T = 0.253$). (Bottom) Dissipation normalised by the laminar value along the same trajectory. Coloured portions of the curve highlight where the two UPOs are being shadowed according to the criteria discussed in the text. Note the trajectory used in this figure is the same one used in figure 5 of the main manuscript.

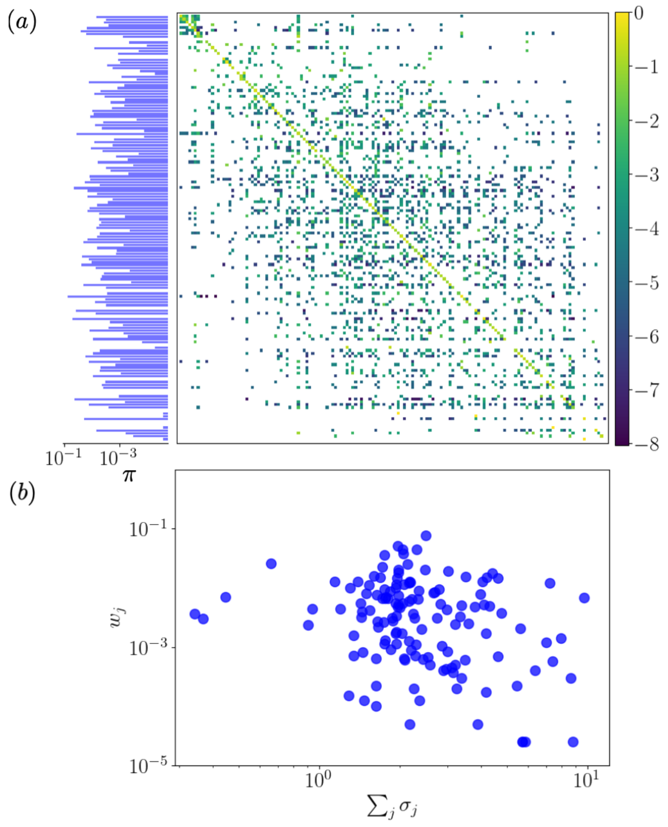

Fig. S2. (a) Invariant measure $\boldsymbol{\pi}$ and transition matrix $\mathbf{P}$ at $Re = 100$ (log of transition probabilities is shown, spacing between snapshots is $\delta t = 0.25$). States are ordered from lowest to highest average dissipation rate (lowest at top/leftmost). (b) The weights in the expansion (7) – which are also the invariant measure of the Markov chain $w_j = \pi_j$ – plotted against the (real part of the) sum of growing Floquet exponents $\sum_j \sigma_j$, $\sigma_j > 0$, for each UPO.

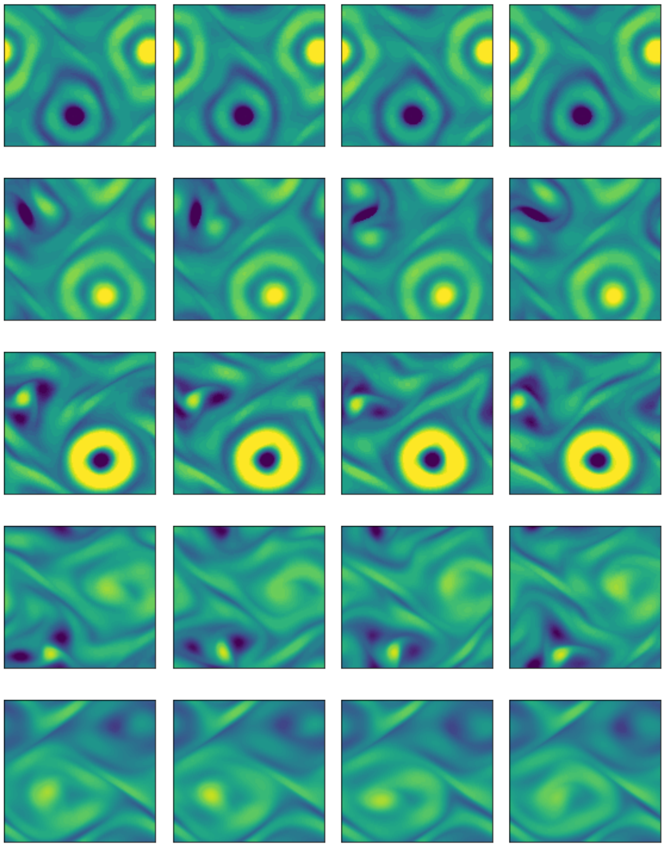

Fig. S3. Spanwise vorticity are extracted at four points equispaced-in-time over five UPOs at $Re = 100$. From top-to-bottom the UPOs have the following periods and average dissipation rates: $(T, \langle D/D_l \rangle) = (1.356, 0.038)$, $(1.723, 0.021)$, $(1.966, 0.068)$, $(2.196, 0.027)$ and $(2.590, 0.02)$ (for full details of converged solutions see table S2). Vorticity contour levels run between ± 10.

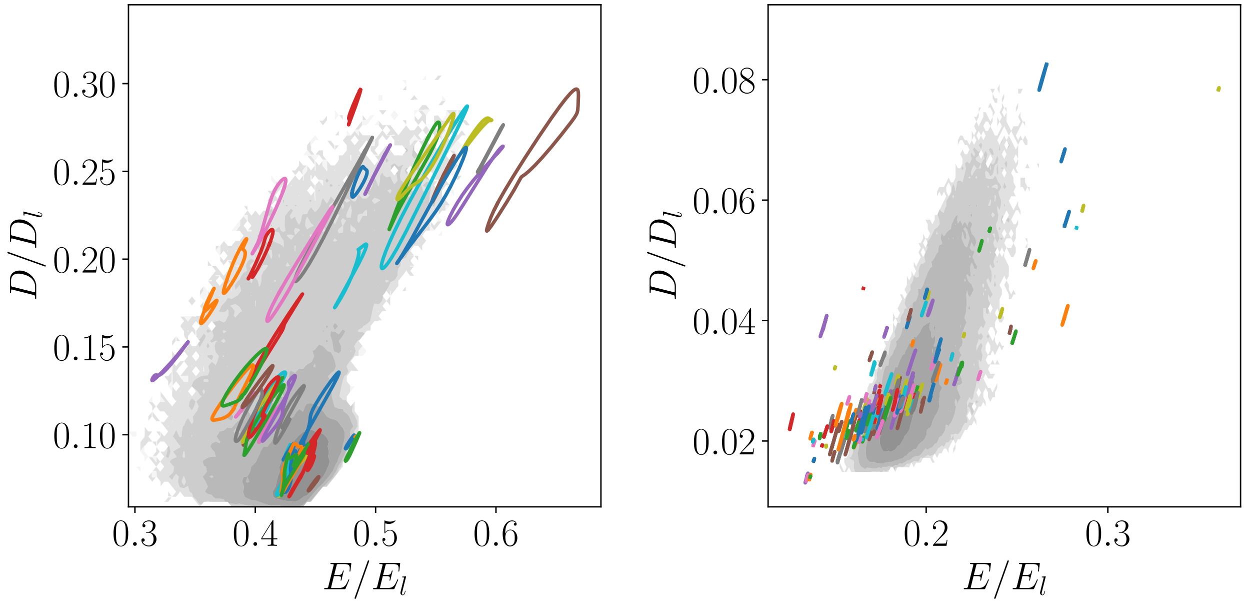

Fig. S4. Kinetic energy against dissipation rate at both $Re = 40$ (left) and $Re = 100$ (right). Grey background is the PDF computed from a long turbulent computation with $T = 2.5 \times 10^5$. Contour levels are spaced logarithmically with a minimum value of 10^{-6}. Closed loops are the two-dimensional projections of 46 converged UPOs. All values are normalised by the laminar values $E_l = Re^2/(4n^4)$ and $D_l = Re/(2n^2)$.

Table S1. Periodic orbits at $Re = 40$. Known solutions as listed in (1) are listed in the 'UPO' column; T is the period, α the shift. N indicates the number of unstable directions and σ_r is the growth rate in the leading Floquet exponent. We also report the average dissipation normalized by the laminar value, $\langle D/D_l \rangle$.

UPO	T	α	N	σ_r	$\langle D/D_l \rangle$
R19	12.207	0.243	2	0.07	0.072
	22.597	0.257	4	0.209	0.081
R24	19.779	0.248	6	0.202	0.081
R6	20.808	0.06	3	0.172	0.083
R22	19.723	0.222	4	0.172	0.083
	18.771	0.457	3	0.105	0.084
R34	23.157	0.265	3	0.113	0.084
P1	5.38	0.0	7	0.191	0.093
P2	2.83	0.0	5	0.223	0.095
P3	2.917	0.0	7	0.236	0.099
	6.523	0.0	6	0.201	0.107
	7.002	0.194	6	1.376	0.108
	7.156	0.032	4	0.292	0.109
R4	6.72	0.106	8	0.343	0.111
	6.352	0.156	6	0.34	0.111
	6.639	0.151	7	0.34	0.113
	6.961	0.0	5	1.386	0.113
	7.735	0.101	7	0.28	0.115
	4.616	0.545	12	2.081	0.115
	8.067	0.742	7	0.243	0.116
	3.452	0.362	11	0.247	0.117
	7.375	0.0	7	0.301	0.118
	7.391	0.081	8	0.219	0.118
	3.689	0.392	8	0.258	0.126
	7.516	0.193	9	0.24	0.127
	6.397	0.439	11	0.19	0.141
	4.706	0.173	10	0.253	0.156
	5.992	0.099	12	0.274	0.173
	5.062	1.324	10	0.253	0.192
	4.6	0.615	10	0.341	0.194
	5.789	0.236	12	0.394	0.199
	5.423	0.372	13	0.348	0.201
	5.241	1.206	11	0.366	0.223
	4.745	0.842	13	0.378	0.223
	3.488	1.142	12	0.444	0.224
	3.749	0.0	11	0.393	0.236
	4.359	0.288	14	0.366	0.243
	2.901	0.486	13	0.356	0.246
	4.15	0.384	15	0.581	0.246
	4.488	1.28	20	0.526	0.247
	3.176	0.424	15	0.504	0.251
	5.234	0.526	15	0.559	0.253
	6.876	0.188	15	0.534	0.254
	3.841	0.945	14	0.497	0.26
	3.396	0.926	18	0.479	0.273
	3.275	0.588	17	0.462	0.285

Table S2. Periodic orbits at $Re = 100$. Known solutions as listed in (1) are listed in the 'UPO' column; T is the period, α the shift. N indicates the number of unstable directions and σ_r is the growth rate in the leading Floquet exponent. We also report the average dissipation normalised by the laminar value, $\langle D/D_l \rangle$.

UPO	T	α	N	σ_r	$\langle D/D_l \rangle$
	4.654	0.073	9	0.098	0.014
R14	4.526	0.071	9	0.105	0.014
	4.58	0.072	7	0.093	0.014
	4.607	0.071	8	0.089	0.014
	4.524	0.023	9	0.386	0.017
	3.34	0.048	10	2.614	0.017
	6.741	0.104	12	0.13	0.018
	2.986	0.525	12	0.294	0.019
	2.976	0.532	15	0.315	0.019
	2.756	0.075	13	0.527	0.02
	2.285	0.435	16	0.359	0.02
	2.695	0.076	8	0.48	0.02
	6.569	0.216	11	1.35	0.02
	2.231	0.427	19	0.365	0.02
	2.241	0.077	11	0.411	0.02
	2.053	0.072	12	0.563	0.02
	2.59	0.067	13	0.539	0.02
	2.594	0.045	15	0.491	0.02
	2.362	0.102	10	0.368	0.021
	2.362	0.465	14	0.392	0.021
	3.943	0.06	17	2.243	0.021
	4.361	0.199	12	0.599	0.021
	2.441	0.002	10	0.347	0.021
	2.442	0.002	10	3.981	0.021
	2.281	0.438	12	0.306	0.021
	2.398	0.12	10	0.472	0.021
	2.601	0.057	18	0.259	0.022
	4.45	0.246	10	0.503	0.022
	2.098	0.118	9	0.297	0.022
	4.669	0.169	9	0.297	0.022
	2.33	0.138	13	0.378	0.022
	2.465	0.052	17	3.682	0.022
	2.281	0.087	9	0.416	0.022
	4.488	0.152	10	0.462	0.022
	5.793	0.065	14	0.218	0.022
	2.527	0.092	12	0.403	0.022
	2.742	0.492	11	0.444	0.022
	2.018	0.553	10	0.399	0.022
	2.176	0.117	14	0.269	0.022
	1.709	0.043	13	0.429	0.022
R16	1.938	0.121	6	0.271	0.023
	2.29	0.013	13	0.394	0.023
	3.536	0.651	15	0.254	0.023
	4.052	0.215	18	0.586	0.023
	2.301	0.037	10	0.429	0.023
	2.777	0.48	17	0.408	0.023
	3.84	0.078	15	2.336	0.023
	3.573	0.363	14	0.272	0.024
	4.422	0.443	15	0.262	0.024
	1.944	0.545	14	0.452	0.024
	4.635	0.386	15	0.26	0.024
	2.61	0.089	16	0.504	0.024

Table S2. Continued

UPO	T	α	N	σ_r	$\langle D/D_l \rangle$
	3.312	0.874	13	0.419	0.024
	2.662	0.12	15	0.542	0.024
	2.592	0.069	13	0.481	0.024
	2.82	0.046	14	0.385	0.025
	1.984	0.122	17	0.556	0.024
	4.321	0.93	14	0.436	0.025
	2.2	0.012	13	0.454	0.025
	5.821	0.089	14	0.318	0.025
	4.497	0.109	15	0.368	0.025
	3.231	0.12	11	0.663	0.025
	4.457	0.644	12	0.293	0.025
	3.359	0.329	12	0.381	0.025
	3.611	0.802	12	2.539	0.025
	4.464	0.079	12	0.408	0.025
	3.736	0.338	9	0.546	0.026
	3.93	0.347	11	0.398	0.026
	3.303	0.385	16	0.423	0.026
	3.829	0.378	13	2.325	0.026
	6.32	0.588	10	0.263	0.026
	1.899	0.299	15	0.323	0.026
	3.919	0.941	15	0.394	0.026
	3.588	0.577	11	0.259	0.026
	5.175	0.138	13	0.616	0.026
	3.869	0.148	13	0.23	0.026
	2.205	0.032	14	4.383	0.026
	3.186	0.836	15	0.43	0.026
	4.139	0.396	10	0.496	0.026
	2.398	0.399	10	0.279	0.026
	4.099	0.533	14	0.312	0.026
	3.736	0.992	16	2.526	0.026
	3.685	1.039	12	0.418	0.027
	2.163	0.382	18	4.197	0.027
	2.196	0.166	13	0.432	0.027
	4.09	0.027	15	0.324	0.027
	3.581	0.169	9	0.243	0.027
	4.162	0.756	15	0.255	0.027
	1.917	0.113	16	0.575	0.027
	2.238	0.346	11	0.328	0.027
R17	3.827	0.008	16	0.818	0.027
	2.136	0.025	11	0.422	0.027
	1.881	0.195	9	0.275	0.027
	3.506	0.094	17	2.639	0.027
	3.993	0.292	15	0.505	0.027
	4.212	0.765	13	0.335	0.027
	3.228	0.407	15	0.406	0.028
	2.688	0.18	21	0.476	0.028
	3.248	0.909	13	0.481	0.028
	1.345	0.007	7	1.113	0.028
	3.757	0.17	11	0.22	0.028
	3.51	0.068	13	2.621	0.028
	3.311	0.885	11	0.417	0.028
	1.328	0.09	24	0.656	0.029
	1.999	0.049	14	0.643	0.029
	4.0	0.003	10	0.32	0.029
	1.178	0.081	22	0.671	0.029
	2.123	0.319	11	0.349	0.029
	3.645	0.169	15	0.249	0.03

Table S2. Continued

UPO	T	α	N	σ_r	$\langle D/D_l \rangle$
	1.711	0.207	8	0.267	0.03
	2.135	0.036	16	0.376	0.03
	1.941	0.425	14	4.804	0.031
	1.861	0.241	18	0.414	0.031
	1.723	0.204	15	0.525	0.031
	3.63	0.561	15	0.364	0.031
	1.729	0.206	14	0.245	0.031
	1.313	0.022	24	0.815	0.032
	3.65	0.037	15	0.992	0.032
	3.774	0.577	16	0.597	0.032
	3.498	0.077	11	0.306	0.033
	3.623	0.063	10	2.572	0.033
	2.031	0.168	10	0.37	0.034
	1.246	0.02	9	1.167	0.034
	1.744	0.014	17	5.067	0.034
	1.757	0.028	14	1.175	0.034
	3.438	0.184	15	2.733	0.035
	1.788	0.085	11	1.034	0.036
	1.71	0.404	12	0.403	0.037
	1.356	0.126	19	0.896	0.038
	1.601	0.051	18	0.505	0.038
	1.718	0.376	14	0.483	0.039
	3.113	0.44	20	0.827	0.039
	2.877	0.056	26	1.229	0.04
	1.993	0.032	23	1.285	0.041
	1.635	0.363	14	0.438	0.041
	1.763	0.111	11	0.439	0.041
	1.746	0.027	20	0.468	0.042
	1.858	0.378	14	0.509	0.042
	1.821	0.082	13	1.142	0.044
	2.005	0.028	15	1.402	0.045
	1.883	0.228	19	0.958	0.045
	1.287	0.163	19	1.037	0.049
	1.425	0.846	12	0.629	0.05
	1.794	0.012	16	1.582	0.053
	1.946	0.0	16	1.615	0.055
	2.658	0.336	14	2.863	0.055
	1.424	0.815	12	6.785	0.057
P4	1.185	0.0	16	1.201	0.059
	1.966	0.435	20	1.536	0.068
	1.164	0.768	14	1.062	0.078
	1.939	0.0	29	1.724	0.08

References

1. GJ Chandler, RR Kerswell, Invariant recurrent solutions embedded in a turbulent two-dimensional Kolmogorov flow. *J. Fluid Mech.* **722**, 554–595 (2013).
